# Machine learning-based risk prediction of postoperative deep vein thrombosis in Chinese patients undergoing gastrointestinal surgery

**DOI:** 10.3389/fcvm.2025.1630099

**Published:** 2025-10-30

**Authors:** Likui Huang, Lihua Gong, Jun Chen, Xiaojing Chen, Bicha Yao, Zhengrong Wang, Shuwei Weng

**Affiliations:** ^1^ Department of Surgery, The First Hospital of Putian City, Affiliated Hospital of Putian University, Putian, Fujian, China; ^2^ Day Ward, Chengxiang District Hospital, Putian, Fujian, China; ^3^ Department of Public Health, The First Hospital of Putian City, Affiliated Hospital of Putian University, Putian, Fujian, China; ^4^ Department of Cardiovascular Medicine, The Second Xiangya Hospital, Central South University, Changsha, Hunan, China; ^5^ Research Institute of Blood Lipid and Atherosclerosis, Changsha, Hunan, China; ^6^ Department of Cardiology, The First Affiliated Hospital of Fujian Medical University, Fuzhou, Fujian, China

**Keywords:** machine learning, deep vein thrombosis, gastrointestinal surgery, Chinese population, risk prediction, clinical prediction model

## Abstract

**Background:**

Deep vein thrombosis (DVT) is a common and potentially life-threatening complication after gastrointestinal surgery. Traditional risk assessment tools rely on static variables and may not effectively capture dynamic perioperative changes.

**Methods:**

Clinical data from 596 Chinese patients undergoing gastrointestinal surgery were retrospectively collected. Patients were randomly divided into training and validation sets (7:3 ratio). Five machine learning algorithms—logistic regression (LR), Extreme Gradient Boosting (XGBoost), multilayer perceptron (MLP), random forest (RF), and elastic net (ENet)—were applied to identify key predictive features and build risk prediction models. The optimal model was visualized using a nomogram and validated through calibration curves, receiver operating characteristic (ROC) curves, and decision curve analysis (DCA).

**Results:**

Among the five models, the RF model achieved the best predictive performance. Postoperative Day-7 D-dimer, Day-1 D-dimer, and Day-5 D-dimer were identified as the most important predictive features. The calibration curve and DCA further confirmed the nomogram's predictive accuracy and clinical utility.

**Conclusion:**

We developed a novel machine learning–based model for predicting postoperative DVT in Chinese patients after gastrointestinal surgery. Integrating dynamic biomarkers and nonlinear modeling, the tool enhances early identification of high-risk individuals. Multicenter validation is warranted to further strengthen the model's applicability.

## 1 Introduction

Venous thromboembolism (VTE), including deep vein thrombosis (DVT) and pulmonary embolism (PE), is a common and severe complication following abdominal surgery (1). Surgical procedures and hospitalization are significant risk factors for VTE, with major surgeries considered independent risk factors due to prolonged immobilization, vascular injury, and increased hypercoagulability induced by surgical stress (2). Among VTE, DVT is the most frequent manifestation, characterized by abnormal blood coagulation within deep veins, lumen obstruction, and subsequent impairment of venous return. Although approximately half of DVT patients may be asymptomatic, nearly one-third are at risk of developing PE, a potentially fatal complication (3).

Systematic reviews have shown that the incidence of perioperative DVT in gastrointestinal surgery generally remains low but varies significantly based on surgical approach and procedure type. Specifically, major open abdominal surgeries, particularly those involving oncologic resections or emergency procedures, carry a higher risk of DVT, whereas minimally invasive techniques have relatively lower risks (4, 5). Current evidence suggests that prophylactic anticoagulant strategies combining mechanical and pharmacological methods effectively reduce postoperative DVT in selected patients (6). However, in patients with very low baseline risk, the hemorrhagic complications associated with routine anticoagulant prophylaxis may outweigh its clinical benefits (5).

Despite these findings, existing risk assessment tools for perioperative DVT remain limited in predictive accuracy and generalizability. Traditional scoring systems, such as the Caprini or Padua scores, primarily rely on static preoperative variables and may not fully capture dynamic perioperative changes in coagulation status, inflammatory response, and hemodynamics (7, 8). Moreover, their applicability to diverse populations, including Chinese patients undergoing gastrointestinal surgery, remains uncertain. Consequently, there is a pressing need for more precise and individualized risk stratification models that integrate a broader range of clinical, laboratory, and procedural data.

At present, there is no robust predictive model specifically designed for assessing the risk of perioperative DVT in Chinese patients undergoing gastrointestinal surgery. To address this gap, we developed a novel machine learning-based prediction model tailored to this patient population. This model incorporates patient demographics, laboratory biomarkers, and perioperative metrics measured at multiple time points. The model aims to facilitate early risk stratification, optimize thromboprophylaxis strategies, and improve overall patient outcomes.

## 2 Materials and methods

### 2.1 Data sources and study population

Between January 2024 and January 2025, clinical data were retrospectively collected from patients who underwent gastrointestinal surgery at The First Hospital of Putian, Fujian Province, China. The study protocol was reviewed and approved by the Ethics Committee of The First Hospital of Putian (Approval No: 2023-121). The data collection window fell entirely within the period covered by this ethics approval. All procedures adhered to the principles outlined in the Declaration of Helsinki and relevant guidelines governing research involving human participants. Given the retrospective nature of the study, the requirement for informed consent was waived.

**Figure 1:**
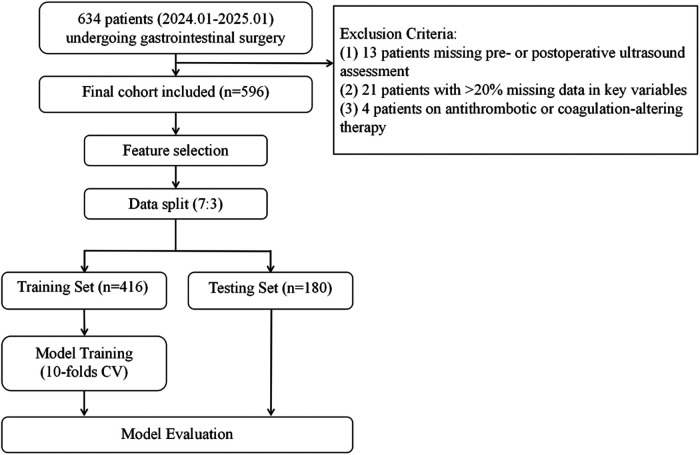
Study flowchart.

Patients were eligible for inclusion if they met all of the following criteria: (1) absence of preoperative deep vein thrombosis confirmed by ultrasonography, with available postoperative ultrasound assessments within seven days following surgery; (2) complete D-dimer measurements at baseline and on postoperative days 1, 3, 5, and 7. For all other critical clinical variables, patients with a per-patient missingness rate >20% were excluded; and (3) no recent use of medications known to influence coagulation or anticoagulation functions. The patient inclusion and modeling process was illustrated in Figure 1.

### 2.2 Diagnostic criteria for DVT

Preoperative and postoperative assessments of deep vein thrombosis were performed using lower extremity venous ultrasonography. All examinations were independently conducted by two qualified attending sonographers from the Department of Ultrasound at The First Hospital of Putian. In cases of discrepancy between the two assessors, the final diagnosis was determined by a senior ultrasound specialist holding the title of associate chief physician or higher. All biochemical tests and physical examinations were completed prior to the ultrasonographic assessments.

Prior to surgery, all patients underwent standardized bilateral lower limb venous ultrasonography to exclude the presence of pre-existing DVT. To ensure consistency in outcome assessment, all patients underwent repeat standardized bilateral lower limb ultrasonography on postoperative Day 7 to detect newly developed DVT. The diagnostic criteria for DVT were based on the international consensus guidelines for ultrasound evaluation (9) and included the following three key findings: (1) incomplete compressibility of the vein under probe pressure; (2) presence of abnormal hyperechoic intraluminal structures suggestive of thrombus; and (3) absence or significant reduction of blood flow signals on color Doppler imaging at the suspected thrombus site. The occurrence of DVT within seven days postoperatively was designated as the primary outcome of the study and served as the basis for model development and risk prediction analyses.

### 2.3 Data preprocessing

A total of 48 clinical parameters were collected from 596 patients, including age, gender, body mass index (BMI), history of alcohol consumption, smoking history, history of infection, preoperative comorbidities such as diabetes, hypertension or other underlying diseases (cardiovascular disease, fracture, and bedrest ≥3 Days), previous surgical history, and history of malignancy. Preoperative clinical data included total cholesterol (TC), triglycerides (TG), low-density lipoprotein (LDL), high-density lipoprotein (HDL), creatinine (Cr), and D-dimer. Postoperative clinical data included TC, TG, LDL, HDL, Cr, D-dimer, C-reactive protein (CRP), D-dimer postoperative Day-1, −3, −5, and −7, systolic blood pressure, and diastolic blood pressure. Surgical and pathological data included American Society of Anesthesiologists (ASA) score, anesthesia method, Fibrinogen, activated partial thromboplastin time (APTT) seconds, prothrombin time (PT) seconds, hemoglobin, platelet count, surgery site, surgery position, temperature, pulse, respiration, blood infusion warming, surgery duration, intraoperative blood loss, internal jugular catheter, and radial artery catheter. Missing data were imputed using the multivariate imputation by chained equations (MICE) method, which models each variable with missing values as a function of the other variables in an iterative manner.

### 2.4 Model development and optimization

Patients were randomly divided into training and testing cohorts at a 7:3 ratio. Five machine learning methods, including Logistic regression (LR), Extreme Gradient Boosting (XGBoost), Multilayer Perceptron (MLP), Random Forest (RF) and Elastic Net (ENet), were used to construct a prediction model for DVT in patients following gastrointestinal surgery. Through ten-fold cross-validation, we determined the optimal hyperparameters of the models (Supplementary Table S1). Additionally, multiple evaluation parameters, including the area under the receiver operating characteristic (ROC) curve (AUC), accuracy, precision, F1-score, and sensitivity, as well as the confusion matrix were used to assess the performance of different machine learning algorithms.

Based on the optimal model, the importance of features was evaluated in the training cohort. Finally, based on selected clinical features, a nomogram was developed using the rms package and validated with the AUC value of ROC, calibration and DCA.

### 2.5 Statistical analysis

All quantitative variables were assessed for their distributional characteristics and were found to be non-normally distributed. Therefore, continuous variables were summarized as medians with interquartile ranges (IQR) and compared between groups using the Wilcoxon rank-sum test. To preserve the completeness of the data and avoid introducing bias, no transformations or manual categorization were applied. All continuous variables were retained in their original scale for statistical comparisons and model development. Categorical variables were expressed as frequencies and percentages and compared between groups using the chi-square (*χ*^2^) test. A two-sided *P*-value < 0.05 was considered statistically significant. Data preprocessing and model construction were performed using R (version 4.1.3), Python (version 3.9.7), and TensorFlow (version 2.5.0). Model training was executed on a workstation equipped with an NVIDIA RTX 3070 Ti GPU, 64 GB RAM, and an 11th Gen Intel(R) Core(TM) i5-11400 @ 2.60 GHz CPU. The operating system was Windows 10 Professional 64-bit (Version 21H1; DirectX 12).

## 3 Results

### 3.1 Baseline characteristics of DVT and non-DVT groups

In terms of baseline characteristics, it was found that age, history of alcohol, FIB, hemoglobin, CRP, preoperative D-dimer, and D-dimer postoperative Day-1, −3, −5, and −7 were statistically different between the patients in the DVT and non-DVT groups (*P* < 0.05, Table 1). Specifically, hemoglobin and history of alcohol were higher in the DVT group compared to non-DVT group, while age, FIB, CRP, preoperative D-dimer, and D-dimer postoperative Day-1, −3, −5, and −7 were higher in DVT group than these of non-DVT group.

**Table 1:** Comparison of patients’ general information.

Characteristics	Subgroup	Positive (*n* = 128)	Negative (*n* = 468)	*P*-value
Age (years)		69.00 [62.75, 74.00]	66.00 [59.00, 71.00]	0.001
Gender	Female	51 (39.8)	182 (38.9)	0.925
	Male	77 (60.2)	286 (61.1)	
BMI (kg/m^2^)		22.45 [20.12, 24.20]	22.50 [20.40, 24.80]	0.285
ASA score	Ⅰ	2 (1.6)	12 (2.6)	0.573
	Ⅱ	76 (59.4)	294 (62.8)	
	Ⅲ	48 (37.5)	159 (34.0)	
	Ⅳ	2 (1.6)	3 (0.6)	
Anesthesia method	GA with Double-Lumen Tube + Epidural	7 (5.5)	28 (6.0)	0.934
	GA with Intubation	22 (17.2)	95 (20.3)	
	GA with Intubation + Epidural	89 (69.5)	310 (66.2)	
	GA with Intubation + Nerve Block	6 (4.7)	23 (4.9)	
	others	4 (3.1)	12 (2.6)	
Surgery site	Abdominal	120 (93.8)	437 (93.4)	0.958
	Others	1 (0.8)	5 (1.1)	
	Thoracoabdominal	7 (5.5)	26 (5.6)	
Surgery position	Lithotomy	10 (7.8)	52 (11.1)	0.517
	Others	7 (5.5)	21 (4.5)	
	Supine	111 (86.7)	395 (84.4)	
Blood infusion warming	No	13 (10.2)	32 (6.8)	0.284
	Yes	115 (89.8)	436 (93.2)	
Internal jugular catheter	Yes	67 (52.3)	203 (43.4)	0.088
	No	61 (47.7)	265 (56.6)	
Radial artery catheter	Yes	118 (92.2)	411 (87.8)	0.219
	No	10 (7.8)	57 (12.2)	
Smoking history	No	119 (93.0)	450 (96.2)	0.195
	Yes	9 (7.0)	18 (3.8)	
Alcohol history	No	125 (97.7)	467 (99.8)	0.045
	Yes	3 (2.3)	1 (0.2)	
Past surgical history	No	86 (67.2)	327 (69.9)	0.635
	Yes	42 (32.8)	141 (30.1)	
History of cardiovascular disease	No	120 (93.8)	438 (93.8)	1
	Yes	8 (6.2)	29 (6.2)	
History of malignancy	No	122 (95.3)	440 (94.2)	0.794
	Yes	6 (4.7)	27 (5.8)	
History of diabetes	No	107 (83.6)	389 (83.1)	1
	Yes	21 (16.4)	79 (16.9)	
History of hypertension	No	86 (67.2)	318 (67.9)	0.955
	Yes	42 (32.8)	150 (32.1)	
History of fracture	No	126 (98.4)	460 (98.3)	1
	Yes	2 (1.6)	8 (1.7)	
History of bedrest	No	126 (98.4)	466 (99.8)	0.229
	Yes	2 (1.6)	1 (0.2)	
History of infection	No	127 (99.2)	458 (97.9)	0.523
	Yes	1 (0.8)	10 (2.1)	
Temperature (℃)		36.50 [36.40, 36.50]	36.50 [36.40, 36.50]	0.932
Pulse (beats per minute)		75.00 [67.00, 82.00]	74.00 [66.75, 83.00]	0.754
Respiration (breaths per minute)		18.00 [18.00, 18.00]	18.00 [18.00, 18.00]	0.083
Surgery duration (hours)		6.53 [5.57, 7.62]	6.00 [5.00, 7.00]	<0.001
Intraoperative blood loss (mL)		100.00 [50.00, 200.00]	100.00 [50.00, 100.00]	0.005
Fibrinogen (g/L)		3.33 [2.88, 3.92]	3.19 [2.70, 3.77]	0.067
APTT (s)		24.55 [22.17, 26.83]	24.80 [22.78, 27.20]	0.333
PT (s)		11.20 [10.80, 11.70]	11.20 [10.80, 11.70]	0.56
Hemoglobin (g/L)		117.50 [100.00, 131.00]	123.00 [109.00, 134.00]	0.01
Platelet Count (×10⁹ /L)		227.50 [177.75, 270.75]	215.00 [178.00, 264.00]	0.586
Preoperative triglycerides (mmol/L)		1.10 [0.83, 1.41]	1.17 [0.86, 1.68]	0.084
Postoperative triglycerides (mmol/L)		0.66 [0.50, 1.15]	0.72 [0.53, 1.12]	0.686
Preoperative total cholesterol (mmol/L)		4.78 [4.20, 5.31]	4.80 [4.05, 5.53]	0.957
Postoperative total cholesterol (mmol/L)		3.72 [3.16, 4.24]	3.71 [3.07, 4.37]	0.827
Preoperative HDL (mmol/L)		1.38 [1.17, 1.65]	1.40 [1.18, 1.63]	0.754
Postoperative HDL (mmol/L)		1.10 [0.94, 1.29]	1.12 [0.96, 1.30]	0.371
Preoperative LDL (mmol/L)		2.79 [2.36, 3.25]	2.81 [2.32, 3.38]	0.939
Postoperative LDL (mmol/L)		2.17 [1.77, 2.52]	2.15 [1.73, 2.56]	0.802
Preoperative Creatinine (μmol/L)		65.50 [55.75, 79.00]	69.00 [55.75, 82.25]	0.213
Postoperative Creatinine (μmol/L)		70.00 [57.00, 83.00]	69.00 [55.75, 82.00]	0.489
Postoperative CRP (mg/L)		37.70 [21.03, 64.20]	25.91 [16.90, 42.40]	<0.001
Preoperative D-Dimer (μg/mL)		0.58 [0.32, 1.16]	0.39 [0.23, 0.79]	<0.001
Postoperative D-Dimer Day1 (μg/mL)		5.48 [3.31, 9.08]	3.20 [1.83, 5.26]	<0.001
Postoperative D-Dimer Day3 (μg/mL)		3.45 [2.26, 5.92]	2.20 [1.41, 3.72]	<0.001
Postoperative D-Dimer Day5 (μg/mL)		3.82 [2.57, 5.98]	2.35 [1.55, 3.69]	<0.001
Postoperative D-Dimer Day7 (μg/mL)		3.93 [2.62, 6.02]	2.01 [1.10, 3.46]	<0.001
Systolic blood pressure (mmHg)		136.00 [126.00, 150.75]	136.00 [124.00, 150.00]	0.338
Diastolic blood pressure (mmHg)		75.00 [69.00, 81.25]	76.00 [69.00, 84.00]	0.337

Continuous variables are presented as median (interquartile range) due to non-normal distributions, and were compared using the Wilcoxon rank-sum test. Categorical variables are presented as counts (percentages) and compared using the chi-square test or Fisher's exact test, as appropriate.

### 3.2 RF model was the optimal model in predicting postoperative DVT

**Figure 2:**
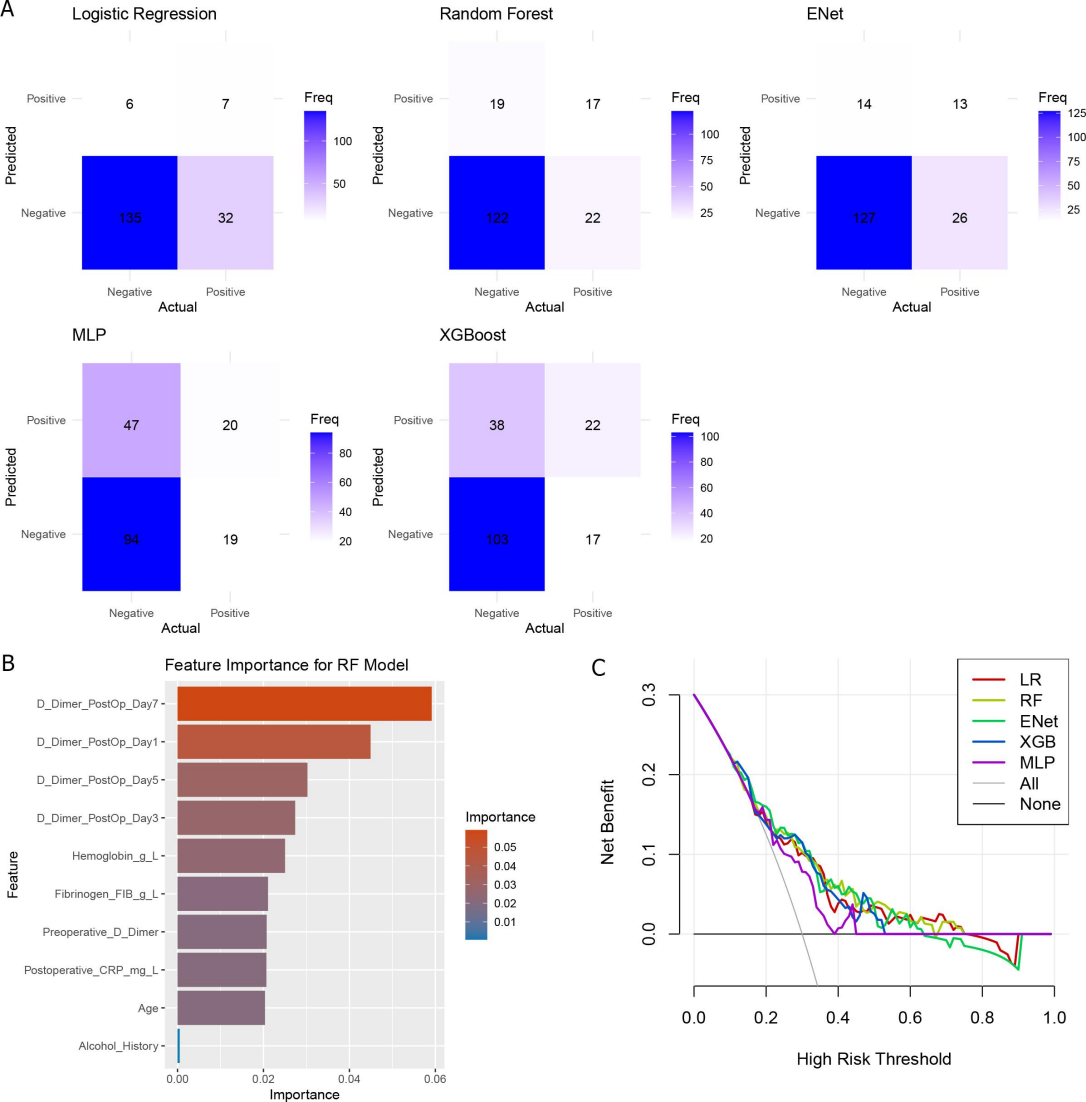
The comparison of machine learning. **(A)** Confusion matrix of five machine learning models; **(B)** Variable importance of random forest models; **(C)** decision curve analysis of five models.

Using the above 10 clinical parameters, we subsequently employed five machine learning algorithms to develop a reliable model for predicting the risk of postoperative DVT. Firstly, patients were divided into training (*N* = 416) and testing (*N* = 180) sets. The training set was used to train different machine learning algorithms, and the testing set was used to evaluate the performance of these algorithms. As shown in Table 2, the AUC values of RF, Elastic Net, and XGBoost were 0.719, 0.728, and 0.720, respectively. But the accuracy of MLP (0.63) and XGBoost (0.69) did not perform well according to the results of confusion matrix (Figure 2A). Finally, RF model was identified as the optimal model for DVT risk prediction and was used for subsequent analysis. To better explain the RF model, the importance of variables in RF was analyzed. We found that D-dimer postoperative Day-7 contributed most, followed by D-dimer postoperative Day-1 and −5 (Figure 2B). Across a range of high-risk threshold probabilities, decision curve analysis demonstrated that all machine learning models provided greater net benefit compared to the “All” and “None” strategies. Among the evaluated models, RF consistently achieved the highest net benefit across most thresholds. LR and ENet exhibited comparable performance, whereas MLP yielded slightly lower net benefits but remained superior to baseline strategies. These findings indicate that RF may offer enhanced clinical utility for risk stratification (Figure 2C).

**Table 2:** Prediction efficiency of different models.

Models	AUC	Accuracy	Sensitivity	Precision	F1 score
Random forest	0.719	0.772	0.847	0.865	0.856
Elastic net	0.728	0.778	0.830	0.901	0.864
Extreme Gradient Boosting	0.720	0.694	0.858	0.730	0.789
Multilayer perceptron	0.689	0.633	0.832	0.667	0.740
Logistic regression	0.673	0.789	0.808	0.957	0.877

### 3.3 A reliable nomogram with good clinical utility was developed for predicting postoperative DVT

**Figure 3:**
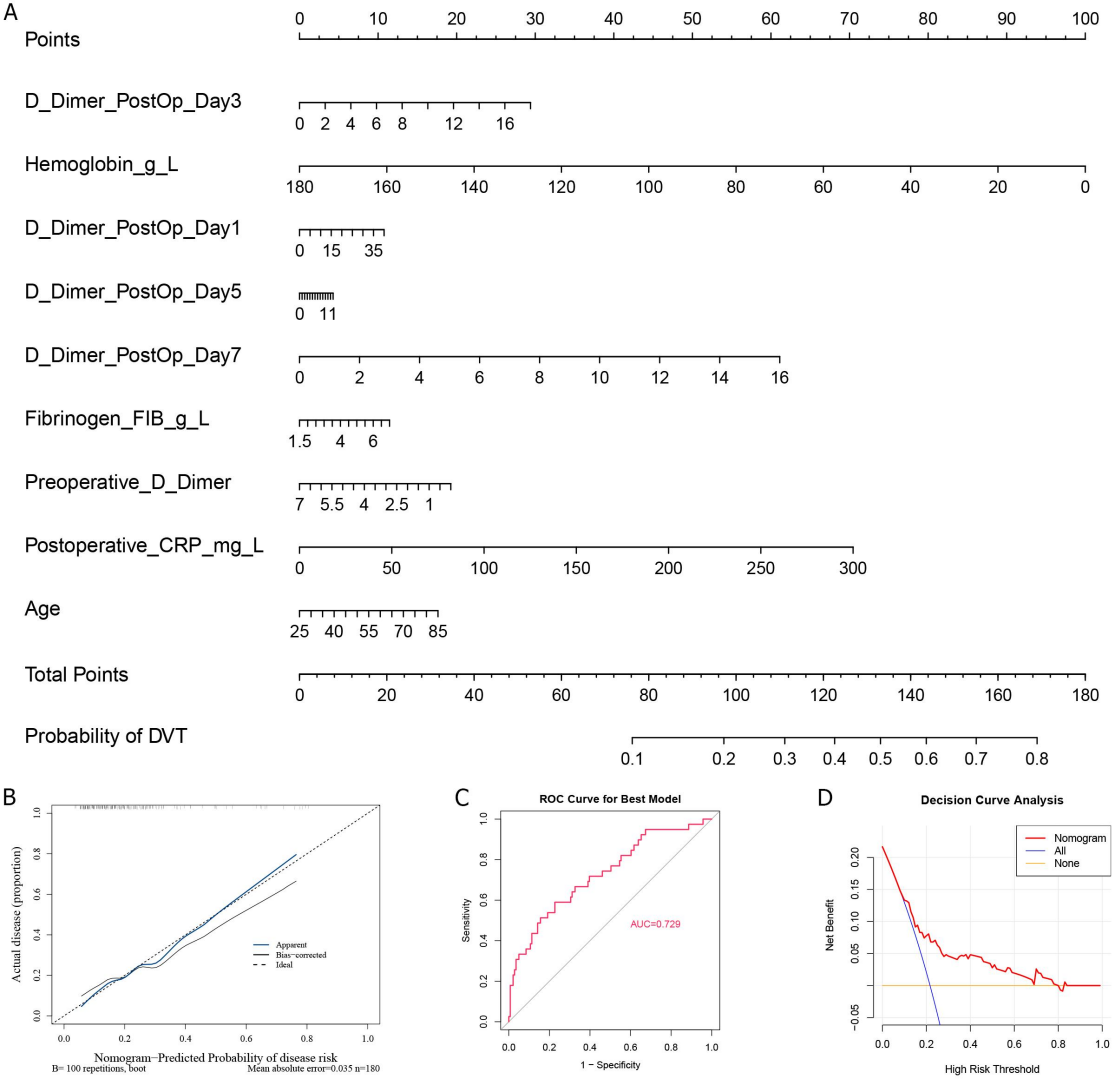
Nomogram establishment and its accuracy analysis. **(A)** Nomogram construction of feature variables; The calibration curve **(B)**, ROC curve **(C)**, and **(D)** decision curve analysis of Nomogram.

To enhance interpretability and facilitate clinical application, we constructed the nomogram using a logistic regression model based on the top-ranked features identified by the Random Forest model. As alcohol history contributed very little to the model, the remaining nine variables were selected for constructing the nomogram, including age, postoperative CRP, preoperative D-dimer, hemoglobin, and D-dimer postoperative Day-1, −3, −5, and −7 (Figure 3A), in which a higher score indicates an increased likelihood of DVT. Furthermore, the calibration curve showed that predicted results were close to actual probabilities (Figure 3B), indicating predicted probability is in good agreement with the actual probability. The AUC value of this nomogram is 0.729 (Figure 3C), further demonstrating its accuracy in predicting DVT. At last, we performed DCA to analyze its clinical utility (Figure 3D), and found that the result of DCA underscored the robust predictive efficiency of the nomogram.

## 4 Discussion

Postoperative DVT is a significant and potentially life-threatening complication in patients undergoing gastrointestinal surgery. Despite the routine implementation of preventive measures, accurately identifying high-risk individuals remains a clinical challenge due to the multifactorial nature of thrombosis and the variability in patient characteristics. Our study aimed to address this issue by developing a machine learning–based predictive model tailored to the Chinese population undergoing gastrointestinal surgery, thereby facilitating early identification of DVT and personalized prevention strategies.

By analyzing clinical data from 596 patients, we constructed and evaluated five machine learning models. Among them, the Random Forest model demonstrated the most balanced and robust predictive performance, with an AUC of 0.719 and relatively high values for precision, sensitivity, and F1-score. It is worth noting, however, that an AUC of 0.719 reflects only moderate discriminatory ability. While this level of performance may be acceptable in a clinical context, further improvements could be achieved by incorporating more granular variables or larger, more diverse datasets. Variable importance analysis identified key predictors: age, fibrinogen, hemoglobin, postoperative CRP, preoperative D-dimer, and D-dimer levels on postoperative days 1, 3, 5 and 7. Compared with traditional risk assessment tools—such as the Caprini Risk Assessment Model, which is widely used to predict VTE risk in surgical patients—our machine learning model offers several critical advantages. Although the Caprini score has been validated and is commonly adopted, it relies on predefined static clinical variables and expert consensus, lacking the ability to incorporate dynamic postoperative biomarkers or capture complex nonlinear interactions among predictors. In contrast, our model integrates both static features and dynamic variables, thereby providing a more comprehensive and individualized risk profile. Furthermore, whereas the Caprini model stratifies patients into broad risk categories, our model yields continuous probability estimates, potentially supporting more nuanced clinical decisions and personalized thromboprophylaxis. These findings highlight the potential of artificial intelligence–driven approaches to complement or even surpass traditional scoring systems in perioperative risk stratification.

Postoperative inflammation and coagulation activation are believed to play key roles in the development of DVT. CRP, as a classic marker of systemic inflammation, reflects the physiological response to surgical trauma and stress. Several prospective studies have demonstrated a strong association between elevated CRP levels and increased VTE risk. For example, Folsom et al. found that individuals in the highest decile of CRP levels had a 1.76-fold higher risk of VTE than those in the lowest decile (10). A meta-analysis by Kunutsor et al. involving 81,625 participants reported that each 5 mg/L increment in CRP was associated with a 23% increased risk of VTE (11). Although Mendelian randomization studies have not confirmed a direct causal relationship between CRP and VTE (12), elevated CRP remains a sensitive indicator of the hypercoagulable state during the postoperative period. Thus, dynamic monitoring of CRP during the perioperative period may aid in early risk identification of DVT.

Simultaneously, D-dimer, a fibrin degradation product, is a well-established biomarker reflecting coagulation and fibrinolytic activity. Khaira et al. reported that D-dimer testing had a sensitivity of 96% and a negative predictive value of 95% for ruling out DVT, significantly reducing the need for invasive venography (13). In the PROLONG study, Palareti et al. found that patients with abnormal D-dimer levels one month after discontinuing anticoagulation had a significantly increased risk of VTE recurrence, supporting the value of serial D-dimer monitoring in postoperative management (14). From a mechanistic perspective, Adam et al. described the sequential enzymatic processes leading to D-dimer formation—namely the actions of thrombin, factor XIIIa, and plasmin on crosslinked fibrin—further reinforcing its biological relevance in thrombosis assessment (15).

Nonetheless, postoperative D-dimer elevations are frequently confounded by surgical trauma and inflammation, particularly in the early postoperative phase, leading to potential false positives (16). To address this, our study incorporated both preoperative and postoperative D-dimer levels to capture dynamic patterns, and employed machine learning to model nonlinear risk evolution over time, thereby improving prediction accuracy. Moreover, several studies have advocated the use of age-adjusted D-dimer thresholds to improve specificity in elderly populations, which may further enhance future model optimization (17).

In addition to CRP and D-dimer, age, fibrinogen, and hemoglobin were also identified in this study as key predictive variables, each of which has shown significant associations with DVT risk through distinct biological mechanisms and supported by growing clinical evidence. Age is a well-established and unmodifiable risk factor for VTE. Advancing age contributes to a prothrombotic state through multiple mechanisms, including endothelial dysfunction, decreased fibrinolytic activity, and the accumulation of comorbid conditions such as immobility, cardiovascular disease, and malignancy (18, 19). A recent prospective study using phenotypic age acceleration from the UK Biobank further confirmed that biological aging significantly increases DVT risk, particularly when combined with genetic susceptibility (20).

Fibrinogen, a pro-coagulant acute-phase reactant in plasma, plays a pivotal role in thrombus formation and stabilization. It is considered valuable for detecting VTE or postoperative DVT, particularly when measured alongside D-dimer levels (21). Multiple studies have demonstrated that elevated postoperative fibrinogen levels are associated with an increased risk of DVT. For instance, a retrospective analysis by Fang et al. involving 842 patients with spontaneous intracerebral hemorrhage confirmed that fibrinogen levels were significantly higher in patients with DVT than those without. Moreover, the combination of fibrinogen, D-dimer, and Caprini score substantially improved DVT prediction accuracy (22). Another study also reported that fibrinogen levels above 4.145 g/L independently predicted the presence of residual venous thrombosis after trauma (23).

Hemoglobin, a key indicator of systemic oxygen-carrying capacity and metabolic status, has also demonstrated potential predictive value in DVT risk stratification. Recent studies suggest that low hemoglobin levels may be associated with increased DVT risk, particularly among high-risk populations such as patients undergoing surgery, trauma, or cancer treatment. A large multicenter study involving 1,596 patients with traumatic fractures identified low hemoglobin as an independent preoperative risk factor for DVT (24). Similarly, in a retrospective analysis of 3,147 ovarian cancer patients, low hemoglobin levels were significantly associated with preoperative DVT and remained statistically significant in multivariate analysis, further supporting the role of hemoglobin as a useful predictive marker (25). Additionally, a study by Fendri et al. observed that approximately one-quarter of patients with DVT also presented with anemia. Some of these cases were accompanied by folate deficiency and hyperhomocysteinemia, potentially contributing to thrombogenesis through endothelial dysfunction and coagulation pathway modulation (26).

To enhance clinical applicability, we translated the RF model into a user-friendly nomogram for bedside risk assessment, bridging the gap between complex modeling techniques and practical decision-making. Decision curve analysis (DCA) further confirmed the model's clinical utility, demonstrating net benefit across a wide range of risk thresholds. This supports the integration of our tool into perioperative workflows to guide personalized thromboprophylaxis strategies, such as tailoring anticoagulation intensity or initiating early mobilization protocols for high-risk patients.

Despite these strengths, several limitations should be acknowledged. First, our study was conducted using data from a single institution, which may limit the generalizability of the model. Although the sample size was relatively large, external validation using multicenter cohorts is essential to confirm model robustness. Second, although we incorporated a broad set of clinical variables, some potentially relevant features—such as genetic predisposition, postoperative mobility, or medication adherence—were not fully captured. Third, the DVT outcome was assessed within a limited postoperative time window using ultrasound, which may underestimate delayed thrombotic events. Fourth, heterogeneity within the study population—such as variations in comorbidities, baseline risk factors, or perioperative management—may have influenced the model's predictive performance across different subgroups. Fifth, the class imbalance between DVT and non-DVT cases may have influenced the model's performance. Although we attempted to mitigate this by applying SMOTENC for resampling, no significant improvement in predictive performance was observed. This suggests that future studies should consider incorporating a larger number of positive DVT cases to enhance model training and robustness. Future studies incorporating real-time monitoring data and expanding the model to account for long-term thromboembolic outcomes may further improve predictive performance and clinical value.

## 5 Conclusion

In conclusion, this study developed and validated a machine learning–based prediction model for assessing the risk of postoperative deep vein thrombosis (DVT) in Chinese patients undergoing gastrointestinal surgery. By integrating static and dynamic clinical variables—particularly serial D-dimer levels and postoperative CRP—the model demonstrated superior predictive performance compared to traditional risk scoring systems. The Random Forest model, translated into a nomogram and supported by decision curve analysis, offers a practical tool for individualized thromboprophylaxis decision-making. Key predictors, including CRP, D-dimer, fibrinogen, and hemoglobin, underscore the central roles of inflammation, coagulation, and metabolic status in DVT pathogenesis. While the findings highlight the promise of data-driven approaches in perioperative risk stratification, external validation and inclusion of additional dynamic and behavioral factors are needed to enhance generalizability and clinical utility in broader populations.

## Data Availability

The datasets presented in this article are not readily available because the raw clinical dataset contains direct personal identifiers such as names, national identification numbers, and hospital admission numbers, as well as other information that could potentially reveal participant identity. Our institutional ethics approval does not allow public sharing of these data. To protect participant privacy and comply with local data protection regulations, we can share a deidentified dataset and the analysis code. Requests to access the datasets should be directed to the corresponding authors after a simple data use agreement and institutional approval.
