# Supplementary material for: Machine learning-based risk prediction of postoperative deep vein thrombosis in Chinese patients undergoing gastrointestinal surgery
Source: Front Cardiovasc Med. 2025 Oct 30;12:1630099. doi: 10.3389/fcvm.2025.1630099 (PMC12613043; doi:10.3389/fcvm.2025.1630099)
Supplement: Supplementary file 1 [file Supplementaryfile1.docx]

**Supplementary Materials**

**Table S1.** Summary of the optimal hyperparameter settings used for each machine learning model.

| **Models** | **The optimal hyperparameter** | |
| --- | --- | --- |
| Logistics Regression | Not applicable | set.seed=123 |
| Random Forest | mtry = 2; trees = 1131; .config = 'Iter10' |  |
| Elastic Net | penalty = 0.1693665; mixture = 0.9030377; .config = 'Iter12' |  |
| EXtreme Gradient Boosting | trees = 1411; tree_depth = 1; learn_rate = 0.00117079; .config = 'Iter24' |  |
| Multilayer Perceptron | hidden_units = 1; epochs = 12; .config = 'Iter2' |  |

**Table S2.** MICE specifications

| **Item** | **Parameter** | |
| --- | --- | --- |
| Number of imputations | m=5 | set.seed=123 |
| Iterations per chain | maxit=10 |  |
| Method | Predictive mean matching (k=5) |  |
| Data leakage control | Imputation performed after the train/test split; the imputation model was fitted on the training set only and then applied to the test set. |  |
| Imputation strategy | Variables with <20% missingness were imputed using MICE; variables with ≥20% missingness were excluded from modeling. |  |
